# Modified Hemilaminectomy for Bilateral Exposure in Intramedullary Spinal Cord Tumor Resection

**DOI:** 10.3390/brainsci16030314

**Published:** 2026-03-16

**Authors:** Sergio Paolini, Anthony Kevin Scafa, Roberta Morace, Vito Chiarella, Rocco Severino, Giuseppe Corazzelli

**Affiliations:** 1 Department of Neurosurgery, IRCCS Neuromed, 86077 Pozzilli, Italy; sergio.paolini@uniroma1.it (S.P.); anthonykevin.scafa@uniroma1.it (A.K.S.); roberta.morace@yahoo.it (R.M.); chiarellavito1@gmail.com (V.C.); 2 Neurosurgery Department, Azienda Ospedaliera Universitaria (AOU) San Giovanni e Ruggi d’Aragona, 84131 Salerno, Italy; severinorocco@gmail.com; 3 Neurosurgery Department, Santa Maria delle Grazie Hospital, ASL Napoli 2 Nord, 80078 Pozzuoli, Italy

**Keywords:** ependymoma, hemangioblastoma, intramedullary, minimally invasive, tumor, unilateral approach

## Abstract

**Highlights:**

**What are the main findings?**

- A modified hemilaminectomy can provide bilateral dorsal spinal cord exposure and allow midline myelotomy through a unilateral posterior approach in selected cases.
- In this series, the technique was associated with satisfactory resection rates and preservation of postoperative neurological function.

**What are the implications of the main findings?**

- This approach may represent a viable minimally invasive option for carefully selected intramedullary spinal cord tumors.
- Preservation of posterior elements and tailored dural management could contribute to limiting postoperative morbidity and maintaining spinal cord–dural separation.

**Abstract:**

**Background:** Posterior approaches to intramedullary spinal cord tumors traditionally rely on bilateral laminotomy or laminoplasty to ensure adequate midline exposure and contralateral dissection. Unilateral approaches are seldom applied in this context, due to concerns regarding insufficient visualization and limited working angles across the midline. **Objective:** To describe a modified hemilaminectomy technique designed to achieve safe midline myelotomy and bilateral tumor dissection through a unilateral corridor, preserving the structural and clinical benefits of minimally invasive posterior access. **Methods:** Fourteen patients with intramedullary spinal cord tumors underwent resection via a refined hemilaminectomy technique, which incorporated systematic thinning of the spinous process and strategic dural suspension. Pre- and postoperative neurological status was assessed using the modified McCormick scale. Surgical parameters, postoperative outcomes, and radiological follow-up were retrospectively collected. **Results:** Gross total resection was achieved in 13 of 14 patients (92.9%), with no new permanent neurological deficits. The mean surgical duration was 194.8 ± 55.9 min, and mean hemoglobin decrease was 1.47 ± 0.94 g/dL. Early postoperative improvement in McCormick grade was observed in 50% of cases, with statistically significant overall functional recovery (*p* = 0.013). No cases of postoperative cord tethering were identified on follow-up magnetic resonance imaging. The approach was technically reproducible and ergonomically favorable, with a shallow learning curve in surgeons experienced with conventional hemilaminectomy. **Conclusions:** The modified hemilaminectomy technique enables effective bilateral exposure and safe midline myelotomy through a unilateral approach, achieving high resection rates with minimal morbidity. It represents a feasible and reproducible alternative to bilateral approaches and warrants prospective validation.

## 1. Introduction

Hemilaminectomy has progressively emerged as a viable alternative to traditional bilateral posterior approaches in a variety of spinal pathologies, including intradural extramedullary tumors, vascular malformations, and degenerative disorders [1,2]. By limiting muscular dissection and preserving midline osteoligamentous structures, this approach has been associated with reduced operative time, lower intraoperative blood loss, diminished postoperative pain, and a decreased risk of progressive spinal deformity, particularly in younger patients and multilevel procedures [3,4].

Despite these recognized advantages, the application of hemilaminectomy to intramedullary spinal cord tumors remains limited and is generally approached with caution [5,6,7]. Adequate visualization of the dorsal columns is essential to ensure safe and accurate placement of the midline myelotomy and to allow meticulous dissection along the contralateral tumor interface [8,9]. Concerns regarding the restricted angle of view, suboptimal control of the surgical midline, and limited access to the contralateral side have traditionally favored the use of bilateral laminotomy or laminoplasty in this setting [10,11,12].

In the present technical note, we describe a modified hemilaminectomy technique designed to overcome these limitations by enhancing midline exposure and bilateral visualization while preserving the structural and clinical benefits of a unilateral approach. The technique involves targeted thinning of non-load-bearing bony elements and strategic dural suspension to create a nearly orthogonal working corridor, allowing safe midline myelotomy and contralateral tumor dissection comparable to bilateral exposure. We report our initial experience in a consecutive series of patients with intramedullary spinal cord tumors and discuss the technical nuances, early outcomes, and potential role of this approach in contemporary spinal oncology.

## 2. Materials and Methods

### 2.1. Patients and Data Collection

The modified hemilaminectomy technique was applied in a consecutive series of adult patients with intramedullary spinal cord tumors deemed suitable for resection through a posterior unilateral approach.

Eligibility was established on preoperative contrast-enhanced MRI and included lesions located at the midline or with a dorsal or eccentric dorsal component, in which the posterior median sulcus remained identifiable, and a safe midline myelotomy was considered feasible [13]. Lesions with predominant ventral extension, circumferential growth pattern, or poorly defined dorsal midline anatomy were excluded, as these conditions were considered unsuitable for safe unilateral access and contralateral dissection [14,15]. All consecutive patients fulfilling these criteria during the study period were included in the analysis. 

**Figure 1:**
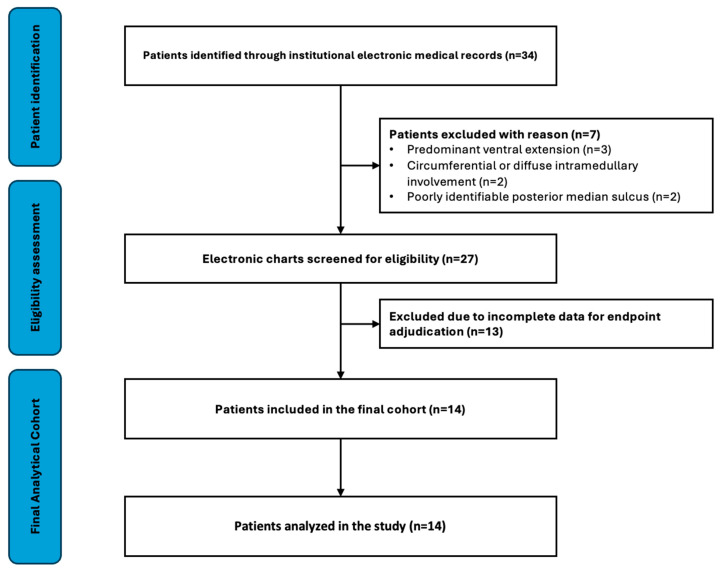
Flow diagram illustrating patient screening, eligibility assessment, exclusions, and final inclusion in the study cohort.

The patient screening and inclusion process is summarized in Figure 1.

Neurological status was evaluated pre- and postoperatively using the modified McCormick scale. Neurological outcomes were assessed during routine clinical follow-up by treating spine neurosurgeons using the modified McCormick scale, according to standard institutional neurological evaluation. Intraoperative neurophysiological monitoring was routinely employed. Postoperative follow-up included both clinical evaluation and spinal MRI. According to institutional practice, MRI was routinely scheduled at approximately 3, 6, 12, and 24 months after surgery, with additional clinical and radiological assessments performed when clinically indicated. Radiological assessment for postoperative tethering was based on the presence of dorsal spinal cord–dural adhesions, defined as loss of the cerebrospinal fluid plane between the dorsal cord and dura, focal dorsal displacement or angulation of the cord, or persistent focal cord flattening at the surgical level. Postoperative imaging was independently reviewed by two spine neurosurgeons not involved in the index surgery. All patients provided written informed consent. The study was conducted in accordance with the Declaration of Helsinki; institutional review board approval was waived due to the retrospective nature and anonymized data. As this study was not a prospective clinical trial, a clinical trial number is not applicable.

### 2.2. Operative Procedure

All procedures were performed under general anesthesia with the patient positioned prone; cranial fixation was applied for cervical lesions. Neurophysiological monitoring included motor-evoked potentials, somatosensory-evoked potentials, free-running electromyography, and, when feasible, D-wave recording.

Following a midline skin incision, unilateral paraspinal muscle dissection was performed to expose the hemilamina. A standard hemilaminectomy and flavectomy were completed. To optimize midline exposure, a systematic modification of the hemilaminectomy was adopted: the base of the spinous process was thinned using a high-speed drill. In cases of bifid spinous processes, the ipsilateral branch was progressively thinned and drilled away; for non-bifid processes, the apex was aggressively tapered. When needed, a shallow groove was created at the tip of the residual spinous process to anchor retraction sutures.

The dura mater was incised along the midline and gently suspended laterally. Contralateral retraction was obtained by anchoring sutures into the paraspinal musculature or lodging them within the pre-drilled groove, thereby exposing the dorsal surface of the spinal cord without the need for bilateral bone removal.

**Figure 2:**
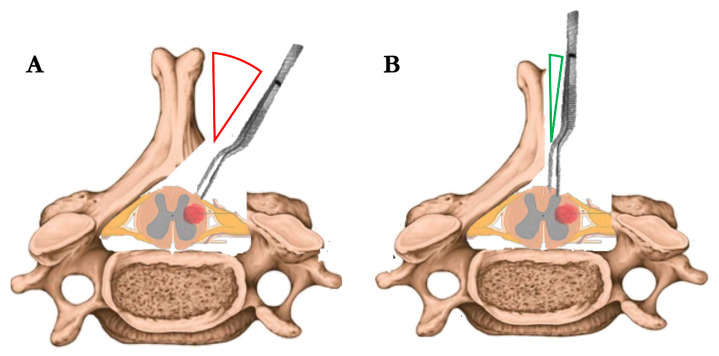
Schematic comparison between a standard unilateral approach (**A**) and the modified unilateral approach with augmented midline exposure (**B**). (**A**) In the standard configuration, the angle of approach to the midline is steep and tangential (red angle), limiting visualization and instrument maneuverability across the contralateral plane. (**B**) In the modified technique, thinning of the spinous process and targeted dural suspension allow for a more orthogonal angle of approach (green angle), enabling safer midline myelotomy and contralateral dissection without bilateral bone removal.

This approach allowed a near-orthogonal inspection of both dorsal columns (Figure 2), and facilitated precise identification of the posterior median sulcus through anatomical landmarks and intraoperative neurophysiological confirmation. A midline myelotomy was performed under high magnification, followed by meticulous dissection of the tumor from the surrounding spinal cord parenchyma. The tumor was gently removed in a piecemeal or ‘en bloc’ fashion, depending on intraoperative findings. Coagulation was employed sparingly and limited to unavoidable situations, always prioritizing the preservation of neural structures. 

Upon completion, the dura was closed primarily using running 6-0 Prolene sutures. Symmetrical tenting sutures were anchored to the spinous process to maintain dural prominence and reduce the risk of postoperative tethering.

A supplementary surgical video demonstrating the key operative steps of the modified hemilaminectomy technique is available in the Supplementary Materials.

### 2.3. Statistical Analysis

Data were collected and organized using Microsoft Excel, version 2021 (Microsoft Corp., Redmond, WA, USA). Statistical analyses were performed using GraphPad Prism, version 10.0.1.02 (GraphPad Software, San Diego, CA, USA). Given the ordinal nature of the modified McCormick scale and the limited sample size, pre- and postoperative scores were compared using the Wilcoxon signed-rank test. Correlations between neurological outcomes and clinical variables were explored using Spearman’s rank correlation coefficient. Continuous variables are reported as mean ± standard deviation, while ordinal variables are additionally presented as median and interquartile range (IQR). A *p*-value < 0.05 was considered statistically significant.

## 3. Results

### 3.1. Cohort Characteristics

During the study period, 34 patients were screened, and 14 meeting anatomical eligibility criteria and documentation requirements were included in the final analysis (Figure 1).

The included patients underwent surgery using the proposed approach between 2016 and 2024 (Table 1). The cohort included 7 males and 7 females, with a mean age of 53.3 ± 18.4 years. Lesions were most frequently located in the cervical spine (n = 7; 50.0%), followed by the thoracic region (n = 4; 28.6%) and the cervicothoracic junction (n = 3; 21.4%). Most tumors extended over two vertebral levels (n = 9), while two lesions spanned four levels.

Preoperative neurological symptoms included motor deficits in 10 patients (71.4%), sensory disturbances in 12 (85.7%), and sphincter dysfunction in 9 (64.3%). The mean preoperative modified McCormick score was 2.79 ± 1.05. Histologically, the series included 6 ependymomas and 5 hemangioblastomas; in addition, one melanocytoma, one cavernous angioma, and one radiologically suspected low-grade neoplasm without histological confirmation were recorded. WHO 2021 [15,16] grading was available in 11 cases, with 6 tumors classified as grade I and 5 as grade II. Clinical and radiological follow-up was available for all patients, with a mean follow-up duration of 46 ± 20 months (range, 11–61 months).

### 3.2. Surgical and Functional Outcomes

Gross total resection (GTR) was achieved in 13 out of 14 cases (92.9%), as confirmed by intraoperative assessment and postoperative imaging. Subtotal resection was performed in a single case involving a cervicothoracic tumor with infiltrative features and indistinct dissection planes.

The mean duration of surgery was 194.8 ± 55.9 min, and the mean postoperative hemoglobin decrease was 1.47 ± 0.94 g/dL. Mean postoperative length of stay was 5.07 ± 1.44 days. No radiological evidence of postoperative dorsal cord tethering, according to predefined imaging criteria, was observed during the available follow-up period. No intraoperative complications or perioperative deaths occurred.

Postoperatively, neurological status remained stable or improved in most patients. Seven individuals (50.0%) experienced an improvement in their McCormick grade at discharge (five with a one-point and two with a two-point gain), four patients (28.6%) remained unchanged. One patient (Patient’s ID: 13; Table 1) experienced transient postoperative neurological worsening, defined as a temporary deterioration in neurological function that resolved approximately one week after surgery without residual deficit. The event was considered likely related to surgical distress and transient spinal cord manipulation.

No major postoperative complications occurred. Minor complications, including cerebrospinal fluid leak, wound infection, or reoperation, were not observed in this series. 

The difference between pre- and postoperative modified McCormick scores remained statistically significant (Wilcoxon signed-rank test, *p* = 0.012). Median McCormick score improved from 3 (IQR 2–3.75) preoperatively to 2 (IQR 2–3) postoperatively.

Postoperative neurological outcomes correlated with age (Spearman’s ρ = 0.66, *p* = 0.01). A strong positive correlation was observed between pre- and postoperative scores (Spearman’s ρ = 0.83, *p* < 0.001) (Table 2).

#### 3.2.1. Illustrative Case (Pt8)

**Figure 3:**
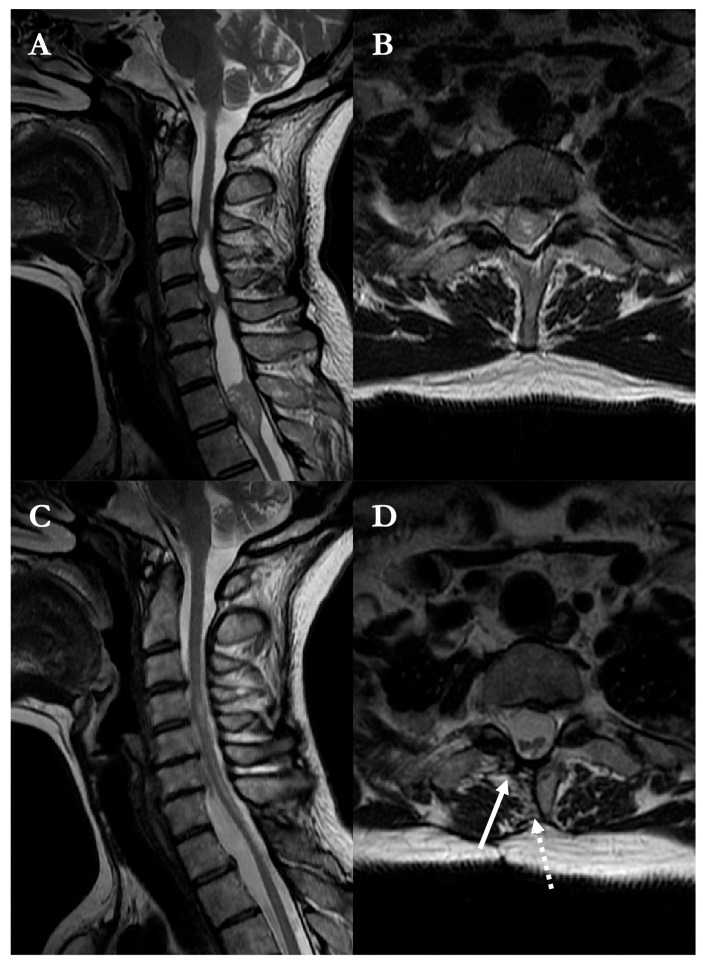
Preoperative and postoperative T2-weighted MRI of the cervical–thoracic spine in the illustrative case 8. (**A**) Sagittal and (**B**) axial views demonstrate a contrast-enhancing intramedullary lesion at the C7–T1 level, associated with syringomyelia extending cranially. (**C**) Postoperative sagittal view at 6-month follow-up confirms complete tumor resection with collapse of the syrinx and restoration of cord morphology. (**D**) Axial view shows a patent midline myelotomy and the modified hemilaminectomy approach (solid arrow) and absence of dorsal tethering, with preserved separation between the spinal cord and dura (dotted arrow).

A 46-year-old woman presented with four-limb paresthesias. MRI demonstrated a contrast-enhancing intramedullary lesion at the T1 level (Figure 3), associated with a syrinx and spinal cord expansion.

**Figure 4:**
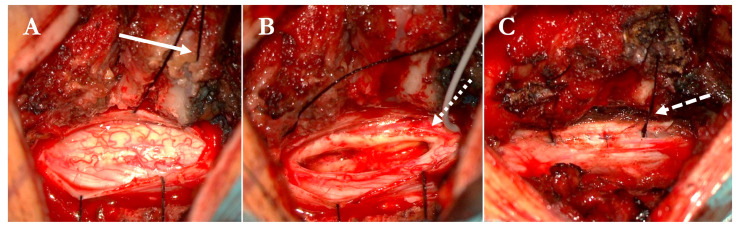
Intraoperative images illustrating key steps of the modified hemilaminectomy technique. (**A**) Unilateral hemilaminectomy with augmented midline exposure reveals the entire dorsal surface of the spinal cord, including the contralateral dorsal root entry zone. The dura is suspended using medial threads anchored into a 2 mm groove drilled within the spinous process (solid arrow). (**B**) Surgical field after tumor removal and midline myelotomy. The dorsal columns are clearly visualized, and the D-wave electrode is positioned for intraoperative monitoring (dotted arrow). No blind spots are present along the contralateral tumor interface. (**C**) Final view after watertight dural closure with running 6-0 prolene suture. Dural tenting is secured by suspending sutures anchored to the spinous process (dashed arrow), contributing to postoperative decompression and potentially reducing the risk of dorsal cord tethering.

A modified hemilaminectomy provided unilateral access to the lesion. Following a midline myelotomy, the tumor was gradually separated from the surrounding spinal cord and resected in a piecemeal fashion under continuous neuromonitoring. Intraoperative photographs document the appearance of the swollen cord, the cleavage plane, and the resection bed (Figure 4). 

Histopathological examination confirmed a WHO grade II ependymoma. The postoperative course was uneventful, and the patient was discharged on postoperative day 5. Follow-up MRI at six months showed complete tumor removal and partial collapse of the syrinx, with restoration of spinal cord anatomy and separation from the dura (Figure 3).

#### 3.2.2. Illustrative Case (Pt14)

**Figure 5:**
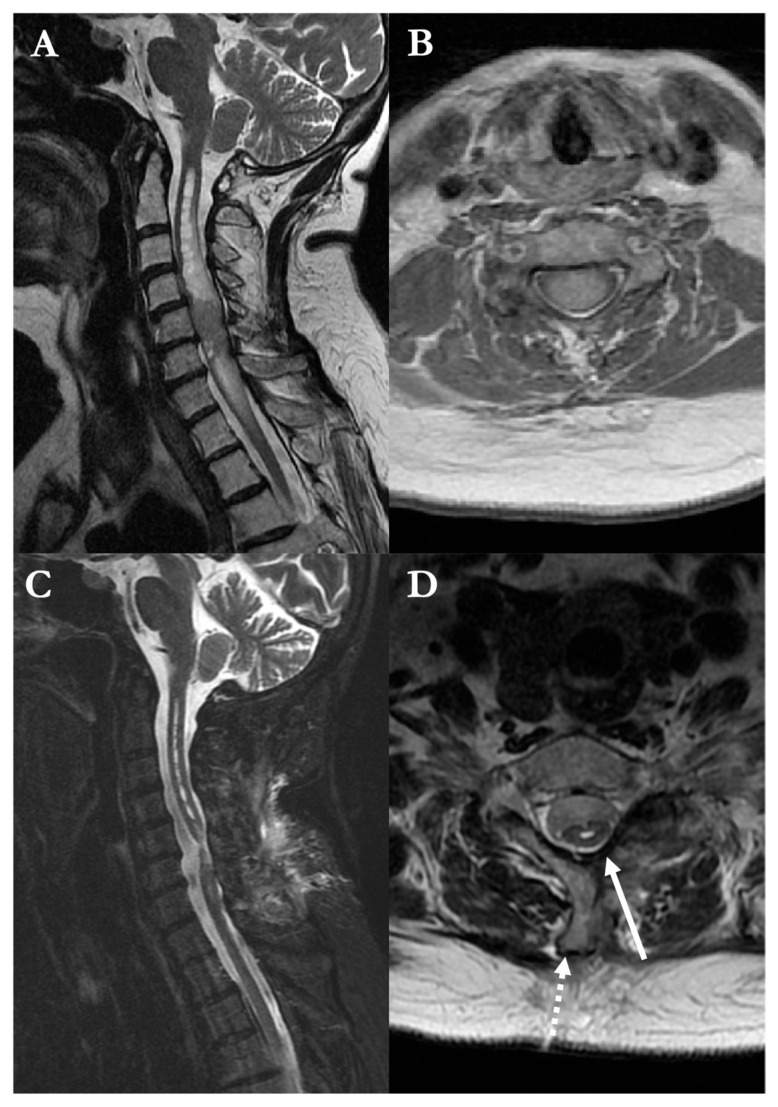
Preoperative and postoperative MRI of the cervical spine in illustrative Case 14. (**A**) Sagittal T2-weighted image shows an expansile intramedullary lesion extending from C4 to C7, associated with spinal cord swelling and blurring of normal anatomical planes. (**B**) Axial contrast-enhanced T1-weighted image demonstrates an ill-defined, heterogeneously enhancing lesion involving the central and right dorsal aspect of the cord, suggestive of a low-grade glioma. (**C**) Postoperative sagittal T2-weighted image demonstrates lesion removal with restoration of spinal cord morphology. (**D**) Axial postoperative T2-weighted image shows the surgical corridor of the modified hemilaminectomy approach (solid arrow) and preserved dorsal cerebrospinal fluid space without evidence of tethering (dotted arrow).

A 66-year-old woman presented with progressive tetraparesis and urinary incontinence. MRI revealed a contrast-enhancing, expansile intramedullary lesion extending from C4 to C7, with associated spinal cord swelling (Figure 5).

Surgical exposure was achieved through a modified hemilaminectomy, followed by midline myelotomy. The lesion appeared poorly circumscribed but was gradually dissected from the surrounding neural tissue under continuous neurophysiological monitoring. Gross total resection was accomplished. Adequate decompression and restoration of spinal cord pulsatility at closure were confirmed by intraoperative inspection. Histological diagnosis was inconclusive, though intraoperative findings and radiological features were suggestive of a low-grade glioma. The patient’s postoperative course was favorable, with improved motor function and resolution of sphincteric symptoms. She was discharged home on postoperative day 4.

## 4. Discussion

Standard approaches to intramedullary spinal cord tumors typically rely on bilateral laminotomy or laminoplasty to ensure sufficient exposure of the dorsal surface [1,10,11,12]. Unilateral approaches, though attractive for their reduced invasiveness and implications on spinal stability, have traditionally been avoided in this setting, due to concerns over limited access [17]. Furthermore, unilateral approaches have been addressed for limited tumor interface, stepwise learning curve, and uncomfortable surgical angles [16,18,19]. A comparative overview of unilateral and bilateral posterior approaches for intramedullary spinal cord tumors was summarized in Table 3.

Our experience with the described modified hemilaminectomy technique advocates that these limitations might be overcome through careful drilling of non-structural bone prominences and strategic dural management [14,38,39]. By thinning the base and apex of the spinous process, especially when bifid [39,40,41], and creating a dedicated groove for suspension sutures, we were able to achieve an operative bilateral exposure, whilst maintaining the structural advantages of a unilateral approach [42,43]. Thinning the spinous process up to its base proved valuable but must be performed cautiously, as excessive drilling may result in intraoperative or delayed fracture [1,43,44]. Notably, the technique was particularly feasible for cervical lesions, where anatomical variations in spinous process morphology may hinder instrument mobility and working angle [12,44,45].

The technique proved safe and potentially reproducible. Admittedly, GTR was achieved in over 90% of cases, with no new permanent neurological deficits and only one case of transient worsening. The mean postoperative McCormick score showed statistically significant improvement, and most patients were discharged within 5 days. Importantly, increasing age correlated with worse postoperative outcomes, reinforcing the need for careful patient selection and intraoperative planning in older individuals.

An unexpected yet promising observation was the absence of postoperative spinal cord tethering in all followed-up patients, during the available clinical and radiological follow-up; however, longer-term evaluation remains necessary, as tethering may represent a delayed complication [46,47,48]. Remarkably, posterior tethering of the spinal cord is a inadequately understood occurrence, generally developing within three months after surgery in 37% to 51% patients undergoing intramedullary tumor resection [49,50,51]. Up to 30% of these patients might develop progressive myelopathy [49,50,51], and asymptomatic patients might experience neurological deterioration in event of revision surgery [47,52]. Currently, no technique has been standardized to prevent dural tethering, due to the poor understanding of this phenomenon [53,54,55]. This technique hypothesizes that preserving the contralateral hemilamina and anchoring dural tenting sutures into the residual interspinous soft tissues may contribute to maintaining dural prominence and potentially preventing fibrous adhesions to neural structures. The described dural closure, along with the bone structures management, might represent a practical alternative to other described reconstruction strategies, as previously proposed.

Furthermore, the learning curve for this technique appears auspicious. Surgeons acquainted with standard hemilaminectomy would find that minor technical modifications—especially related to bone work and dural management—may be rapidly integrated into their routine, with minimal adaptation required in terms of orientation or workflow [18,42,43]. The proposed technique proved useful and safe, as it provided functionally bilateral exposure and allowed contralateral tumor dissection from a nearly orthogonal, rather than tangential, perspective, a critical aspect in our opinion [4,5,6,9,56].

Overall, the approach allowed for confident tumor resection with favorable visualization and handling of the midline and contralateral structures, while preserving the biomechanical and clinical advantages of hemilaminectomy. Although this is not a comparative or prospective study, our findings support the feasibility of the technique even in multilevel or centrally located intramedullary tumors. 

Building upon the present technical report, which aimed to delineate the rationale, execution, and preliminary outcomes of a modified hemilaminectomy approach for intramedullary spinal cord tumor resection, future endeavors will focus on expanding the patient cohort and enhancing the methodological rigor. Specifically, we plan to conduct a prospective, matched case–control study to compare this technique with traditional bilateral approaches, evaluating parameters such as neurological recovery, complication rates, and extent of resection.

Additionally, we intend to apply this approach to cases of tumor recurrence, where minimizing tissue disruption and preserving posterior structures may offer significant benefits. A comprehensive longitudinal assessment will be undertaken to monitor symptom progression, spinal stability, and the incidence of delayed complications, particularly focusing on dural tethering. This phenomenon, often underrecognized, can lead to progressive neurological deficits, including motor and sensory disturbances, as well as bladder and bowel dysfunctions [1,12,49,51,57,58]. By systematically evaluating these aspects in a larger cohort, we aim to elucidate the clinical manifestations and impact of dural tethering post-surgery.

Future studies are expected to provide a more comprehensive understanding of the long-term safety and efficacy of the proposed approach. Ultimately, our goal is to further evaluate whether the modified hemilaminectomy technique may represent a valuable posterior option for selected non-meningeal intramedullary tumors, particularly in anatomically challenging or multilevel cases where both optimal exposure and preservation of spinal integrity are paramount. 

As a technical note, this study is inherently limited by its small sample size and relatively short follow-up, which restricts broader generalization and do not allow assessment of long-term outcomes. Noteworthy, the present work was conceived as a technical feasibility case series and was not designed as a comparative study against standard bilateral approaches. Therefore, the reported outcomes should be interpreted as exploratory and descriptive, rather than as evidence of superiority or comparative efficacy. Future prospective comparative studies will be necessary to determine whether the technical advantages observed in this series translate into measurable clinical benefits. 

Although clinical follow-up was available, longitudinal functional assessment using standardized time points was not uniformly available due to the retrospective nature of the study; therefore, neurological outcomes were primarily reported at discharge as the most consistently documented time point. In addition, the heterogeneity in tumor histology and spinal levels involved may introduce variability in surgical exposure and clinical results. However, intramedullary spinal cord tumors represent a rare group of lesions, and the present work was conceived as a technical note aimed at describing a surgical technique for a specific anatomical subtype of intramedullary tumors rather than performing pathology-specific outcome comparisons. These limitations underscore the need for future case–control studies with larger, stratified cohorts to confirm the reproducibility and clinical value of the proposed technique.

The present experience suggests that targeted technical refinements of the hemilaminectomy approach may offer adequate exposure for intramedullary tumor resection, while maintaining the structural and clinical advantages of a unilateral access. The absence of major complications, together with encouraging functional outcomes and the lack of postoperative cord tethering, supports further investigation of this technique in larger and methodologically structured studies.

## 5. Conclusions

The modified hemilaminectomy approach provided effective bilateral exposure for midline myelotomy while preserving the structural benefits of unilateral access. The technique was associated with minimal blood loss, a favorable learning curve, and consistent reproducibility. No major complications or cases of postoperative tethering were observed. Although limited by sample size and follow-up, these findings support further prospective and comparative studies to assess the broader applicability of this approach in intramedullary tumor surgery.

## Figures and Tables

**Table 1:** Patient demographics, tumor characteristics, and surgical details.

Pt ID	Age (Years)	Sex	Level	Levels Involved	Preoperative Deficit	Histology	2021 WHO Grade	Hb Decrease (g/dL)	Surgery Duration (Minutes)	Preoperative McCormick Score	Postoperative McCormick Score	Hospital Stay (Days)
**1**	45	M	C6–C7	2	Tetraparesis, urinary symptoms	Ependymoma	2	2.3	290	3	2	3
**2**	28	M	C4	1	Balance issues, mild upper limb weakness	Hemangioblastoma	1	1.9	115	2	2	4
**3**	38	M	C7–T1	2	Mild gait disturbance, urgency	Hemangioblastoma	1	1.5	150	3	2	6
**4**	81	M	C7–T1	2	Tetraparesis, urinary urgency	Ependymoma	2	0.4	218	3	3	6
**5**	33	F	C3	1	Right hand sensory deficit, urinary urgency	Hemangioblastoma	1	2.4	125	2	1	4
**6**	31	F	C4–C5	2	Mild sensory deficit, mild balance issues	Ependymoma	2	0.5	180	2	1	7
**7**	67	M	T3–T4	2	Mild lower limb weakness, urinary urgency	Melanocytoma	3	0.8	245	2	2	5
**8**	45	F	T1	1	Lower limb weakness, urinary urgency	Ependymoma	2	1.9	280	2	2	5
**9**	39	F	T10–T11	2	Asymptomatic (small lesion)	Hemangioblastoma	1	1.9	155	1	1	3
**10**	70	M	C7–T3	4	Complete paraplegia, urinary retention	Ependymoma	1	1.5	194	5	5	7
**11**	54	F	C2–C3	2	Severe right-hand weakness, gait disturbance	Intramedullary Cavernous Angioma	-	1.6	165	3	3	6
**12**	75	F	T8–T9	2	Severe paraparesis, urinary retention	Hemangioblastoma	1	0.6	145	4	3	4
**13**	74	M	C6–C7	2	Hand weakness, gait instability	Ependymoma	2	3.1	230	3	3	7
**14**	66	F	C4–C7	4	Tetraparesis, urinary incontinence	Astrocytoma	2	1.4	235	4	2	4

**Table 2:** Summary of cohort characteristics and surgical outcomes.

Variable	Value
Age (years)	53.29 ± 18.44
Sex (M)	7 (50.0%)
Location	7 (50.0%)
Cervical	
Thoracic	4 (28.6%)
Cervicothoracic	3 (21.4%)
Levels involved	
1	3
2	9
4	2
Preoperative McCormick Score	2.79 ± 1.05
Postoperative McCormick Score	2.29 ± 1.07
Preoperative deficit	
Motor	10 (71.4%)
Sensory	12 (85.7%)
Sphincteric	9 (64.3%)
Histology	
Ependymomas	6
Hemangioblastomas	5
2021 WHO grade	
II	5
I	6
Hospital Stay (days)	5.07 ± 1.44
Hemoglobin Drop (g/dL)	1.47 ± 0.94
Surgery Duration (minutes)	194.79 ± 55.92

**Table 3:** Comparative overview of unilateral and bilateral posterior approaches for intramedullary spinal cord tumors.

Variable	Unilateral Approach	Bilateral Approach (Laminectomy/Laminoplasty)
Surgical exposure	Adequate exposure has been reported in selected intramedullary lesions, particularly when tumors are dorsally or eccentrically located and occupy a limited portion of the spinal canal [20,21,22,23].	Provides wide bilateral exposure and direct midline access, facilitating multilevel surgery and unrestricted visualization of the spinal cord [11,24].
Contralateral visualization	Achieved through angled microscopic visualization and contralateral dissection via a unilateral corridor; feasibility depends on surgical expertise and anatomical selection [25,26,27].	Intrinsic bilateral visualization without need for angled working corridors, allowing straightforward contralateral access [11,24,28].
Muscle and posterior element preservation	Contralateral musculature and posterior ligamentous complex are preserved, limiting soft-tissue disruption [20,23,26].	Bilateral muscle detachment and removal of posterior elements may disrupt the posterior tension band [29,30,31,32].
Postoperative spinal stability/deformity risk	Preservation of posterior structures has been described as potentially protective against postoperative instability in selected cases [20,21,22].	Laminectomy has been associated with increased risk of postoperative kyphotic deformity; laminoplasty may reduce, but not eliminate, this risk [11,24,30,33].
Biomechanical impact	Limited unilateral bone removal has been described as preserving biomechanical integrity compared with wider posterior element removal [20,22,29].	Biomechanical studies demonstrate increased motion and reduced stability after laminectomy compared with reconstructive techniques such as laminoplasty [29].
Postoperative complications	Low complication rates have been reported in selected series using unilateral corridors, with limited soft-tissue exposure [21,25,34].	Higher rates of postoperative complications such as CSF leakage and deformity have been reported after extensive bilateral exposure compared with reconstructive approaches [11,30,35].
Need for reconstruction or fixation	Usually not required due to preservation of posterior elements and unilateral bone removal [20,26,36].	Laminoplasty aims to restore posterior anatomy; laminectomy alone may require secondary stabilization in selected cases [24,28,30].
Technical complexity	Requires familiarity with unilateral corridors, angled visualization, and contralateral manipulation; considered technically demanding [25,26,36].	Technically familiar and widely adopted approach with direct exposure and conventional microsurgical ergonomics [11,24,31].
Patient selection	Recommended for carefully selected lesions with favorable dorsal or eccentric location and suitable anatomical corridors [21,26,36].	Applicable to a broader spectrum of intramedullary lesions regardless of lateralization or ventral extension [11,24,37].
Evidence base	Primarily technical notes and small retrospective series focused on feasibility and surgical refinement [20,21,25,28].	Larger retrospective cohorts and meta-analyses available, mainly comparing laminectomy and laminoplasty outcomes [11,30,33].

## Data Availability

The data presented in this study are available on request from the corresponding author due to privacy, legal, and ethical reasons.

## Supplementary Materials

The following supporting information can be downloaded at: https://www.mdpi.com/article/10.3390/brainsci16030314/s1, Video S1: Key operative steps of the modified hemilaminectomy approach for intramedullary spinal cord tumor resection.

## Abbreviations

The following abbreviations are used in this manuscript:

MRI	Magnetic Resonance Imaging
GTR	Gross Total Resection
WHO	World Health Organization
MEPs	Motor Evoked Potentials
SSEPs	Somatosensory Evoked Potentials
EMG	Electromyography
D-wave	Descending corticospinal tract wave
SD	Standard Deviation
IRCCS	Istituto di Ricovero e Cura a Carattere Scientifico
AOU	Azienda Ospedaliera Universitaria
ASL	Azienda Sanitaria Locale
CT	Computed Tomography
